# An updated overview of some factors that influence the biological effects of nanoparticles

**DOI:** 10.3389/fbioe.2023.1254861

**Published:** 2023-08-30

**Authors:** Yang Xuan, Wenliang Zhang, Xinjiang Zhu, Shubiao Zhang

**Affiliations:** ^1^ Key Laboratory of Biological Resources and Utilization of Ministry of Education, Dalian Minzu University, Dalian, Liaoning, China; ^2^ Liaoning Cancer Hospital & Institute, Cancer Hospital of Dalian University of Technology, Dalian, Liaoning, China

**Keywords:** nanoparticles, biological effects, oxidative stress, interaction, protein corona

## Abstract

Nanoparticles (NPs) can be extremely effective in the early diagnosis and treatment of cancer due to their properties. The nanotechnology industry is developing rapidly. The number of multifunctional NPs has increased in the market and hundreds of NPs are in various stages of preclinical and clinical development. Thus, the mechanism underlying the effects of NPs on biological systems has received much attention. After NPs enter the body, they interact with plasma proteins, tumour cell receptors, and small biological molecules. This interaction is closely related to the size, shape, chemical composition and surface modification properties of NPs. In this review, the effects of the size, shape, chemical composition and surface modification of NPs on the biological effects of NPs were summarised, including the mechanism through which NPs enter cells, the resulting oxidative stress response, and the interaction with proteins. This review of the biological effects of NPs can not only provide theoretical support for the preparation of safer and more efficient NPs but also lay the foundation for their clinical application.

## Graphical Abstract

**Figure:**
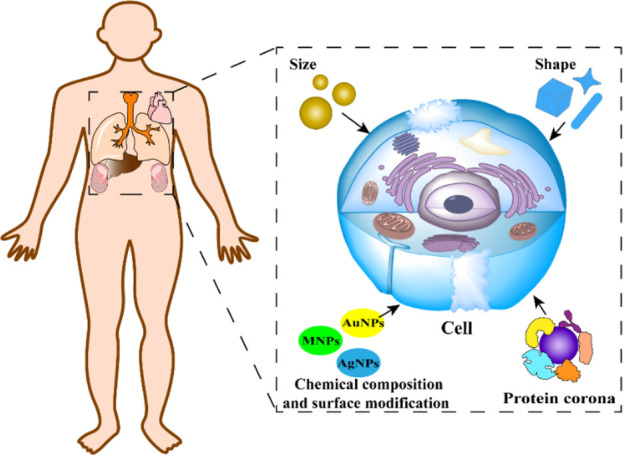
Effects on cells of NPs with different properties.

## 1 Introduction

Cancer is a serious threat to humans. In 2020, more than 19 million people were diagnosed with cancer globally. Nearly 10 million of them died because of cancer. The number of confirmed cases and deaths due to cancer is estimated to reach approximately 28 million and 16 million by 2040, respectively (Mao et al., 2022). The large number of cancer-related deaths is mainly because of the lack of effective cancer detection methods (Fitzgerald et al., 2022), which increases the difficulty in the treatment of cancer. Cancer treatment faces several challenges, including high rates of cancer returning and spreading to other parts of the body (metastasis), significant side effects, and a low cure rate. One important consideration is the development of side effects from chemotherapy, which can lead to discomfort such as nausea, vomiting, hair loss, and fatigue, thereby reducing the patient’s quality of life (Muth, 2017). Additionally, chemotherapy’s toxic effects can cause long-term damage to healthy cells, leading to other health problems. Another concern is that tumor cells might become resistant to drugs, reducing the effectiveness of treatment and overall response rates (Vander Velde et al., 2020). Other conventional treatments like surgical removal or radiotherapy also have their own limitations. Therefore, safe and simple diagnosis and treatment techniques need to be developed to solve the problems and limitations of cancer therapy (Zhang Y. et al., 2022).

Nanotechnology has developed rapidly over several decades (Singh et al., 2021; Hamimed et al., 2022). The design and synthesis of novel NPs may be used in many fields, such as tissue engineering, drug delivery, biological detection, optics, manufacturing, national defence, etc., (Díaz-Caballero et al., 2018; Eivazzadeh-Keihan et al., 2022; Majumder et al., 2022; Rial et al., 2022). For instance, Lee et al. (2017) demonstrated that a delivery mechanism made of AuNPs linked with DNA and combined with cationic polymers that disrupt endosomes could effectively transport the Cas9 ribonucleoprotein and donor DNA to various cell types. They successfully corrected the DNA mutation responsible for Duchenne muscular dystrophy in mice through local injection, resulting in minimal off-target DNA damage. In addition to biomedical applications, NPs have a significant role in catalytic chemistry and other domains. Designing broad-spectrum photocatalysts to capture photons within the visible-light spectrum and augment solar energy conversion remains a daunting task. To tackle this challenge, a hybrid co-catalyst system was devised by Gao et al. using the standard polymeric carbon nitride. This system integrates plasmonic AuNPs with atomically dispersed platinum single atoms, each performing distinct functions. This work proposed a novel approach to craft broad-spectrum photocatalysts for energy conversion reactions (Gao et al., 2023). Notably, among the uses of these NPs, their role in the diagnosis and treatment of tumors is the most recognized (Ho et al., 2019; Janjua et al., 2021; Llop and Lammers, 2021; Shan et al., 2022; Tao et al., 2023). Zhang et al. (2018) developed NPs (Ag_2_S@DP-FA) with high stability, fluorescence imaging capability, and high photothermal conversion efficiency for the early diagnosis and treatment of tumours. Their findings showed that Ag_2_S@DP-FA could effectively target tumours *in vitro* and *in vivo* and improve imaging ability. Thus, these NPs might be used for the diagnosis and treatment of tumours. Yang et al. (2023) developed multifunctional NPs (UF@PPDF). When used along with the targeting molecule and chemotherapeutic drugs, the NPs could perform multifunctional tumour diagnosis and treatment. As these NPs had a multilayer iron oxide structure, the size and position of tumour tissue could be prominently displayed through the dynamic switching between T2 and T1-weighted imaging. Additionally, these NPs had a high photothermal conversion efficiency and could release iron ions and drugs quickly, which enhanced the effectiveness of chemotherapy, photothermal therapy (PTT), and ferroptosis synergistic treatment of tumours. Thus, high-resolution diagnosis and multifunctional therapy (Bendjador et al., 2022) could be performed simultaneously. Yin et al. (2023) designed and synthesised multifunctional NPs (Gd_2_O_3_@Ir/MTB-RVG29). These NPs used the rabies virus glycotide-29 (RVG29) peptide to cross the blood-brain barrier. The researchers also used Ir nanozymes as an intelligent switch to enhance the photothermal effect of NPs in the tumour microenvironment. The Ir nanozymes could also remove excess reactive oxygen species (ROS) and protect normal brain tissue. Due to the paramagnetic properties of Gd_2_O_3_, the NPs could also be used as a magnetic resonance imaging contrast agent for the visualisation and real-time detection of the PTT effect. This technology could be used for the diagnosis and treatment of mouse brain glioma, and it laid the foundation for the clinical application of NPs (Cai et al., 2023).

Although NPs have various applications, their side effects cannot be ignored (Xiong et al., 2022; Stepankova et al., 2023). Nanomaterials can have harmful effects on biological systems due to their small size and distinctive surface properties. Furthermore, when nanomaterials are released into the environment, they can endanger ecosystems because of their lasting presence (Cui et al., 2017). NPs and biomacromolecules have a similar size. They can be easily transported into cells and have high penetration and interaction with cell membranes and tissues. The effects of free radicals generated by NPs in the body or ions generated after decomposition on ion channels in the plasma membrane also need to be investigated. Therefore, the biological effects of NPs, especially their side effects, cannot be ignored. In 2004, Eva Oberdörster from Duke University found that fullerene (C_60_) could lead to significant oxidative damage to fish brains, and subsequent studies showed that C_60_ could also affect the expression of some genes related to the immune response in fish (Oberdörster, 2004; Oberdörster et al., 2005). Zhu et al. found that iron oxide NPs can induce the production of ROS, which can cause further damage to DNA. The induction of cytotoxicity and the inhibition of human aortic endothelial cell activity were found to affect the endothelial system, resulting in atherosclerosis in mice (Zhu et al., 2011). Murphy et al. studied the toxic effects of gold nanoparticles (AuNPs) on human dermal fibroblasts and found that the morphology and gene expression of cells changed significantly (Falagan-Lotsch et al., 2016) when the concentration of AuNPs was 0.1 nM. Additionally, the phagocytosis of NPs by certain cells (for example, phagocytic cells in the immune system) may lead to the release of inflammatory medium-induced cell death, and even cause the death of other cells (Hsiao et al., 2017). NPs can also interact directly with various biomolecules in the body, such as proteins, DNA, and RNA, which can greatly affect their biological functions (Martínez-Negro et al., 2021).

Besides toxic and side effects, the biological effects of NPs might also influence the body’s immune response (Michael et al., 2022), oxidative stress (Lin et al., 2022), modes of cellular uptake (Sun et al., 2023), and metabolism. A comprehensive study on biological effects can not only elucidate the mechanism by which NPs affect cells, organs, and other biological tissues *in vivo*, but it can also lay the foundation for designing safer and more efficient NPs. Such information can help overcome the limitations of the clinical application of NPs and provide new methods for the diagnosis and treatment of cancer and other major diseases. NPs have various biological effects, which depend on their unique physical and chemical properties. The most basic elements include the size, shape, chemical composition, and surface modification of NPs. Additionally, the interaction between NPs and proteins can also influence the biological effects of NPs.

In this review, we discussed the impact of NPs on biological systems, focusing on their primary physicochemical characteristics. We begin by reviewing how size, shape, chemical makeup, and surface alterations of NPs influence their interactions with living entities. This is explored across organ/tissue, cellular, and molecular dimensions from a toxicological viewpoint. We further discuss how surface chemistry plays a pivotal role in protein corona creation and investigate the ramifications of this protein corona on cellular absorption, toxicity, and cellular reactions. Proteins, through their interactions, can modulate the attributes and functions of NPs. Furthermore, we shed light on the reasons and mechanisms behind NP toxicity, outline the obstacles encountered in the realm of nanotoxicology, and suggest strategies to develop NPs with reduced toxicity. We hope our review serves as a theoretical bedrock for the enhanced clinical utilization of NPs.

## 2 Size

When NPs have a similar shape, the change in size might lead to subtle changes in physical and chemical parameters (Le Bars et al., 2022), which can result in changes in the kinetics of NPs (Duncan and Gaspar, 2011). The change in size can affect their half-life in blood (Matsumoto et al., 2009), uptake by immune cells (Sharma et al., 2022a; Sharma et al., 2022b; Yang et al., 2022), metabolism in the kidneys (Wang G. et al., 2022), escape from the reticuloendothelial system, and other factors (Adamczyk et al., 2022). Additionally, the size of NPs determines the size-specific surface area (Huang et al., 2016) and the process by which they enter cells. NPs enter cells through phagocytosis and pinocytosis, which are the major mechanisms of endocytosis. Larger NPs (500 nm) are preferentially taken up via phagocytosis, whereas smaller NPs are taken up more commonly via pinocytosis (Zhao and Stenzel, 2018).

In general, smaller NPs display higher toxic potential than larger ones because smaller NPs have a larger surface area (Zhang F. et al., 2022), which can enhance the catalytic capability between the cell membrane and NPs (Feng et al., 2022). Additionally, smaller NPs may cause greater damage to the structure of the electronic configuration and crystal plane, which might lead to an increase in the reactive surface sites, ROS levels, and other adverse effects. ROS are important signal transducers and can control many intracellular processes (Lennicke and Cochemé, 2021). As signals are involved in normal cell growth and homeostasis pathways, an increase in ROS levels in cells might lead to molecular damage, causing oxidative stress (Sies and Jones, 2020). When non-cellular factors, such as the size, surface, and composition are present, cellular reactions such as the interaction between NPs and cells, mitochondrial respiration, immune response, and ROS-mediated damage can lead to intracellular oxidative stress reactions of NPs, resulting in cytotoxicity (Manke et al., 2013). Therefore, oxidative stress is an important process through which NPs can induce cell damage (Li S. et al., 2022; Hanson and Batchelor, 2022).

Silver nanoparticles (AgNPs) are usually used as an antibacterial agent (Jian et al., 2022). However, the parameters that affect the toxicity of AgNPs have not been fully elucidated by scientists. Zapór et al. studied the cytotoxic effects of AgNPs with different particle sizes (10, 40, and 100 nm). They found that smaller AgNPs caused mitochondrial dysfunction and increased cell membrane permeability mediated by the development of oxidative stress (Zapór, 2016). AgNPs can lead to the overproduction of ROS even at a low concentration and result in strong cytotoxicity and oxidative damage to DNA (Souza et al., 2020).

Several researchers have studied AuNPs (Bodelón et al., 2017) because they have a high electron density (Ozcicek et al., 2021), dielectric properties (Cao et al., 2021), catalytic effect (Lee and Link, 2021), and the ability to bind to various biological macromolecules without affecting their activity and physicochemical properties. The size of AuNPs strongly influences their performance and application range (Bhamidipati and Fabris, 2017; Miao et al., 2018) as it can directly affect the surface curvature (Wy et al., 2022), selectivity (Wu et al., 2022), and catalytic activity (Gerken et al., 2022) of AuNPs. AuNPs can also affect the signalling pathway of cells.

**FIGURE 1:**
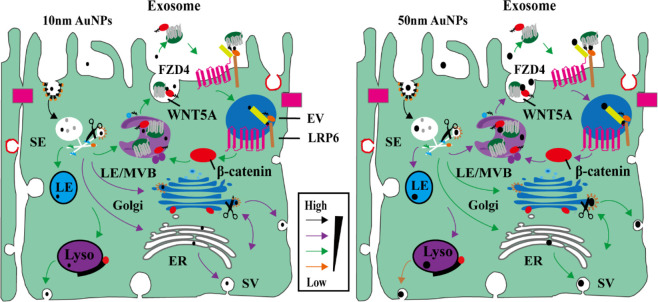
AuNPs size-dependent Wnt/β-catenin signaling pathways in epithelial cells transport pathways (Zhang et al., 2021). Copyright 2021 American Chemical Society.

The association between Wnt/β-catenin signalling and the transport of AuNPs was uncovered by scientists. The researchers meticulously studied the mechanisms of endocytosis and transcytosis of AuNPs by employing pharmacological inhibition strategies (Zhang et al., 2021). They ascertained that the process of cells ingesting AuNPs via endocytosis, as well as their discharge through exocytosis or transcytosis, was controlled by the Wnt signalling pathway, as depicted in Figure 1. Moreover, the researchers determined that larger NPs demonstrated a distinctive desmoid effect, engaging with the Wnt/Frizzled receptor to establish feedback mechanism involving exosomes. This established feedback mechanism subsequently encouraged the expulsion of NPs via exocytosis, while simultaneously diminishing their intake via endocytosis. Consequently, the regulation’s efficacy was found to be contingent upon the size of the NPs. It was also discerned that the nanoparticle size influenced both their longevity in circulation and their distribution within organisms. These findings imply that the size-dependent regulation of nanoparticle transport, guided by the Wnt signalling pathway, may grant valuable insights into nanoparticle behaviour within biological systems. Moreover, it may aid in refining their usage across diverse applications.

The underlying mechanism of interaction between AuNPs and biological systems was also studied by other research groups (Kariuki et al., 2022). Xia et al. (2019) studied the effects of size on the biological distribution of AuNPs *in vivo*. The uptake and cytotoxicity of AuNPs in normal and cancer cells were also evaluated. The results showed that the uptake of AuNPs by cancer cells (HepG2) increased with an increase in their size, whereas the opposite pattern was found in normal cells (L02). Smaller (5 nm) AuNPs showed higher cytotoxicity than larger AuNPs (20 nm and 50 nm) in both cancer and normal cells. The smaller AuNPs (5 nm) induced apoptosis and necrosis of HepG2 cells by stimulating ROS production and the activation of pro-caspase3, whereas it mainly induced necrosis of L02 cells by stimulating the overexpression of TLR2 and the release of IL-6 and IL-1a. The large AuNPs (50 nm) had the longest circulation time and the maximum accumulation in the liver and spleen. However, the 5 nm AuNPs showed higher neutrophils and milder liver toxicity in mice than the 20 nm and 50 nm AuNPs. The results showed that cell uptake, cytotoxicity, and underlying mechanism, as well as, the distribution of AuNPs *in vivo* were all related to the size of AuNPs, which even played a key role in many aspects. Thus, when considering AuNPs for biomedical applications, their size should be accounted for.

In another study, 5 nm and 15 nm AuNPs inhibited cell proliferation through apoptosis and induced chromosome damage, especially chromosome mis-segregation. Compared to the 5 nm AuNPs, the 15 nm AuNPs caused more severe genotoxic effects. The cytotoxicity of AuNPs was found to be independently correlated with their absolute number or tested mass concentration (Di Bucchianico et al., 2014).

Deng et al. (2019) developed a physiologically-based pharmacokinetic model to study the biological distribution of AuNPs of different sizes that were coated with polyethylene glycol. They found that smaller AuNPs had a larger distribution range in organs and a longer circulation time. In most organs, the 24-h accumulation of AuNPs decreased with an increase in the particle size, which indicated that the size of AuNPs played an important role in their distribution and accumulation in various tissues. AuNPs of different sizes accumulate at different locations in organisms, resulting in differences in the distribution of toxicity. However, the toxicity measured by MTT *in vitro* showed that when the dose of AuNPs reached a certain concentration (1 mM), the toxicity increased with a decrease in the size of the AuNPs(Deng et al., 2019). Subsequently, ROS analysis was performed to further study the corresponding mechanism of toxic effects, and the results showed that AuNPs of different sizes could induce different degrees of oxidative stress, which was the main cause of toxicity (Li et al., 2018).

## 3 Shape

NPs can be synthesised in different shapes (Cortés et al., 2021), which can be categorised as nanosphere, nanorod, nanostar, nanowire, nanosheet, nanotube, nanocube, etc. (Sukhanova et al., 2018; Demir, 2021). When NPs enter organisms, their shape can influence the molecular dynamics (Joshi et al., 2022) and *in vivo* toxicity via different cell phagocytosis pathways (Shao et al., 2017).

Using molecular dynamics simulations, Luo et al. (2018) found that the position of NPs passing through the pulmonary surfactant layer was affected by the shape of the NPs. The results showed that hydrophilic NPs with all dimensions smaller than 5 nm could readily penetrate through the pulmonary surfactant layer, and they were not affected by the shape of the NPs. However, the shape played a key role in the translocation and pulmonary surfactant perturbations of larger NPs. As the size of NPs increased, their efficiency of translocation decreased, and the shape of NPs strongly influenced their translocation. NPs that were cuboid, tetrahedral, or had some other shape, and had at least one sharp corner or a needle-like shape, could readily penetrate the pulmonary surfactant layer with a slight pulmonary surfactant perturbation. NPs of other shapes showed lower efficiency of transportation, as characterised by the distance between NPs and the pulmonary surfactant layer. These NPs induced more severe pulmonary surfactant perturbation.

Titanium dioxide nanoparticles (TiO_2_ NPs) are commonly used for treating humans (Xiang et al., 2022). Paolo et al. synthesised TiO_2_ NPs of different shapes (bipyramids, plates, and rods) to study their biodistribution, accumulation, and toxicity after the TiO_2_ NPs were intravenously administered to healthy mice. They found that the accumulation of TiO_2_ NPs was quite low in most organs (except the lungs). Additionally, bipyramidal and plate-shaped NPs showed a higher accumulation, whereas rod-shaped NPs were the most toxic and led to histopathological pulmonary alterations. They could even cause a transient increase in serum markers associated with hepatocellular injury. Overall, small differences in the shape of NPs could substantially alter their accumulation and safety (Dell'Aglio et al., 2023).

Many studies have also investigated the biological effects of AuNPs (Han et al., 2021; Xu et al., 2022; Horwath et al., 2023). Talamini et al. reported that the shape of AuNPs can greatly influence the kinetics of accumulation and excretion of AuNPs in filter organs. They found that when gold nanospheres (AuNSPs), gold nanorods (AuNRs), and gold nanostars (AuNSs) were ingested, AuNSPs and AuNSs showed the same percentage of accumulation, but their localisation in the liver was different, and only AuNSs accumulated in the lungs. The results showed that the kinetic data of the three types of AuNPs that were 50 nm in size were similar in the filter organs (Talamini et al., 2017). However, the accumulation of AuNSs and AuNSPs in the liver and spleen was different from the accumulation of AuNRs (Ma et al., 2013). As the aspect ratio of AuNRs increased, their distribution in the liver and renal excretion decreased, resulting in organ toxicity. Based on the biological distribution of AuNPs and the cytotoxicity mediated by them, the researchers found that the toxicity of AuNPs of different shapes in biomedical applications was different.

Nunes et al. investigated the shape-dependent effects of AuNPs on mitochondrial bioenergetics by exposing isolated rat liver mitochondria to AuNPs of different shapes. The results showed that the presence of AuNRs and AuNSPs led to a decrease in mitochondrial oxygen consumption even in the presence of cyclosporin A, an inhibitor of mitochondrial permeability transition pore. The difference was that AuNRs induced early dissipation of the mitochondrial membrane potential, whereas, AuNSs induced its delay when the same concentration of AuNRs and AuNSs were used (Nunes et al., 2022).

**FIGURE 2:**
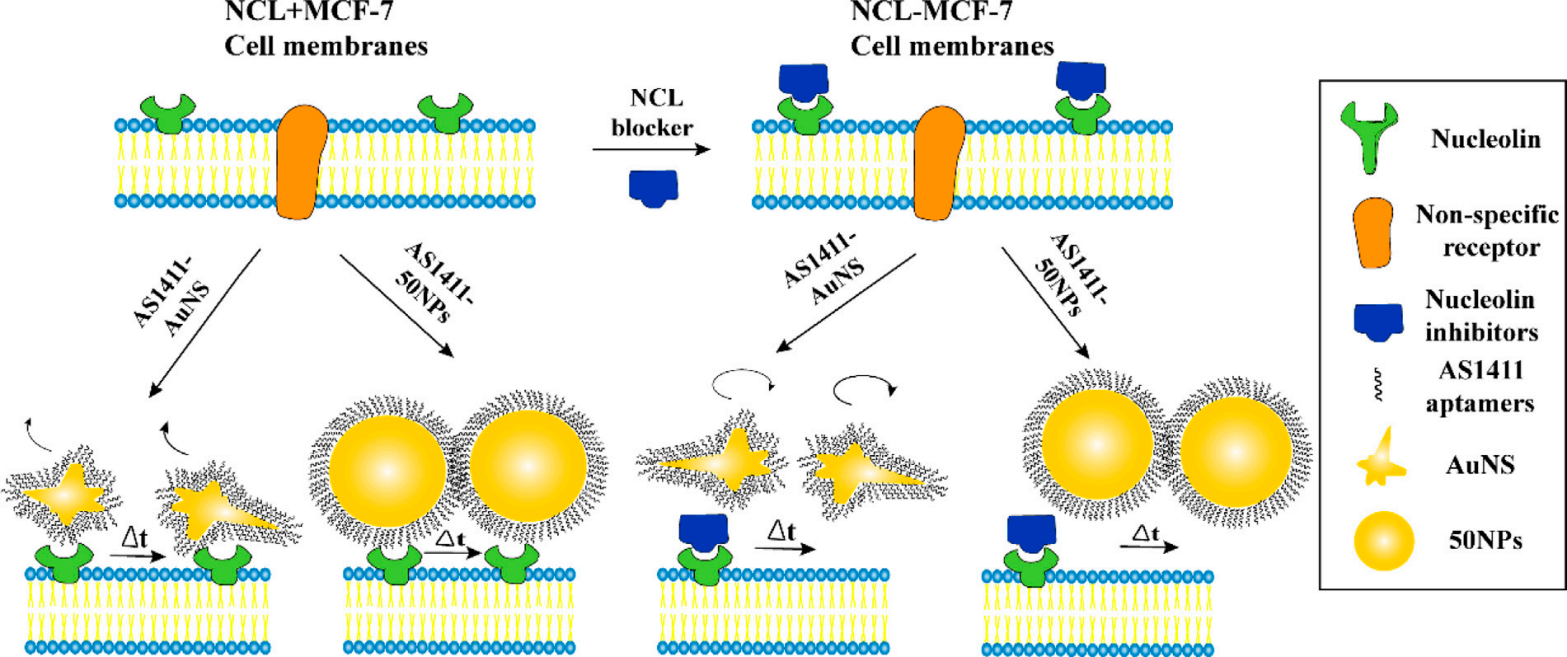
Two nano constructs AS1411-AuNS and AS1411-50 NPs have different targeting specificities for NCL (Choo et al., 2021). Copyright 2021 American Chemical Society.

Choo et al. introduced a new AuNS variant named AS1411-AuNS and AuNSPs (AS1411–50NPs). Using real-time single-particle tracking combined with DIC-epifluorescence imaging, they attached these NPs to a DNA aptamer known as AS1411, enabling them to monitor the NPs' intracellular movements. The main objective was to determine the impact of NP shape on their behaviour within the cell membrane, as depicted in Figure 2. From their study, it became evident that the configuration of the NPs was pivotal in dictating their motion within the cell membrane. The shape of the NPs consistently influenced how the ligand (AS1411) engaged with the receptor on the cell membrane, regardless of the presence or absence of the nucleolin membrane receptors. Such interactions subsequently determined how the NPs targeted these membrane receptors. These results underscore the significance of the nanoparticle’s shape in the realm of targeted delivery system design and in understanding how NPs and cells interact. The specificity and efficacy of therapies and diagnostic methods based on NPs might be considerably affected by these shape-dependent ligand-receptor relationships (Choo et al., 2021). Delving deeper into this subject could pave the way for advancing more precise and efficient nanomedicine strategies (Cho et al., 2010).

Different shapes of NPs can lead to exposure to different crystallite surfaces (Tu et al., 2020), which can result in different degrees of particle membrane association (Spadea et al., 2022). Tree-Udom et al. synthesised Yb^3+^ and Tm^3+^-doped NaYF_4_ NPs of different shapes (sphere, elongated sphere, and hexagonal prism). All three shapes of NPs had the same properties, crystal structure, emission spectrum, and negative surface charge range in water. The degree of particle membrane association between different NaYF_4_ NPs was investigated by using artificial lipid bilayer membrane liposomes that were as large as cells. The results showed that elongated spherical NaYF_4_ NPs bound to lipid bilayer membranes more efficiently than spherical and hexagonal prismatic NaYF_4_ NPs. This occurred because the free energy of the curved membrane around the elongated spherical NPs was lower than that of other shapes. The cytotoxicity of NPs was directly affected by the degree of binding between NPs and the membrane (Tree-Udom et al., 2015).

Besides metallic NPs, the influence of the shape of non-metallic NPs on biological effects was also investigated. Champion et al. investigated the phagocytosis of alveolar macrophages by using polystyrene NPs of different shapes and found that all shapes could initiate phagocytosis in at least one orientation and play a leading role in the phagocytosis of macrophages. The local shape determined the complexity of the actin structure through phagocytosis and allowed the NPs to move across the cell membrane. Their findings indicated that the more complex the shape of the NPs, the less efficient the phagocytosis produced (Champion and Mitragotri, 2006).

## 4 Chemical composition and surface modification

NPs with unique properties are widely used in various fields (Liu et al., 2022), such as industry, agriculture, and biomedicine. The applications of magnetic NPs (MNPs) (Liu et al., 2019), semiconductor NPs (Kment et al., 2017), carbon NPs (Li et al., 2021), and AuNPs (Yang et al., 2015) were found to be extremely important, but different material compositions were found to be associated with different toxicity mechanisms *in vivo*.

Magnetic NPs (MNPs) are unique because they possess magnetism and other specific characteristics (Benayas et al., 2023; Qiao et al., 2023). For example, after modifying target molecules, MNPs could reach the anticipated site to facilitate magnetic resonance imaging, drug delivery, photothermal therapy, etc. (Di et al., 2020). As MNPs can combine with the cytoskeleton after being endocytosed by the cell, the performance of cell mechanics can be changed, which can lead to an increase in cell rigidity. To a certain extent, MNPs can decrease cell proliferation, harm the actin cytoskeleton and microtubule network architecture, and enhance adhesion plaque formation and maturation (Soenen et al., 2010). Several studies have suggested that NPs can also disrupt glucose and energy homeostasis. Shin et al. experimentally showed that silica-coated magnetic NPs containing rhodamine B [MNPs@SiO_2_ (RITC)] were associated with glucose metabolism disorders. At high concentrations, MNPs@SiO_2_ (RITC) could significantly decrease intracellular glucose content and play a key role in reducing the efficiency of glucose uptake. In the metabolic transcriptome network, an increase in ROS levels and impairments in glucose metabolism could lead to a decrease in the efficiency of glucose uptake. These results suggested that MNPs@SiO_2_ (RITC) were associated with ROS production and impaired glucose metabolism (Shin et al., 2019).

Several studies have shown that doping metals in semiconductor TiO_2_ NPs can help in adjusting their optical and electrical properties, which can increase the effectiveness of these NPs in the biomedical field (Ahamed et al., 2016). However, metal-doped TiO_2_ NPs might induce cytotoxicity, inflammation, oxidative stress, and apoptosis (Kongseng et al., 2016). Ahmad et al. reported that Cu-mixed TiO_2_ NPs can induce toxicity and oxidative stress reactions in some tumour cells. When A549 cells were exposed to pure TiO_2_ NPs or Cu-mixed TiO_2_ NPs, the potential of the mitochondrial membrane decreased and the activity of the caspase-3 enzyme increased. Additionally, the cytotoxicity of the Cu-mixed TiO_2_ NPs was higher than that of pure TiO_2_ NPs (Ahmad et al., 2017).

**FIGURE 3:**
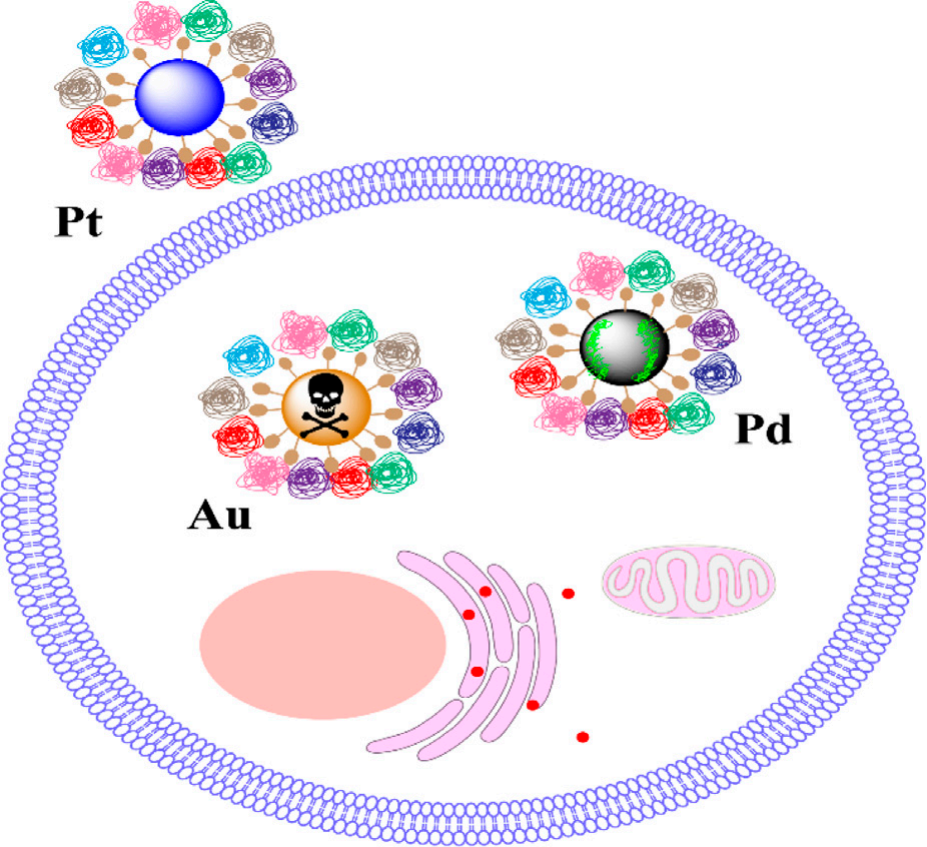
The core material induces cytotoxicity by regulating the levels of CRA (Bai et al., 2020). Copyright 2020 American Chemical Society.

A research initiative compared three NPs - Au, Pt, and Pd - which were identical in size, shape, and surface ligands. The team conducted experiments to understand how these particles were internalised by cells and also assessed cellular ROS measured as HO-1/β-actin) and cytotoxic effects. They found that PtNPs had reduced cellular uptake, which could be attributed to their enhanced hydrophilicity. Moreover, the composition of the material influenced Cellular Redox Activity (CRA) and elicited varying cytotoxic responses. Specifically, PdNPs substantially reduced intracellular H_2_O_2_ concentrations, thereby aiding in cell survival. On the other hand, AuNPs led to elevated levels of cellular oxidative stress and increased cytotoxicity, as illustrated in Figure 3. In essence, variations in CRA and cytotoxicity based on material composition were consistent with observations linked to surface ligands (Bai et al., 2020).

Besides the original material composition of NPs, different surface modifications can also cause different biological effects. Diaz et al. synthesised AuNSPs coated with citric acid (CA) and tannic acid (TA) to compare the effects of surface ligands on blood adsorption and uptake by A549 cells. The surface charge data of both types of AuNSPs exposed to serum proteins showed significant protein adsorption. The results of light scattering experiments indicated that the volume and density of AuNSPs-CA-protein composites were smaller than those of AuNSPs-TA-protein composites. Additionally, the uptake of NPs in cells showed certain differences, the AuNSPs-TA formed small clusters and induced low uptake. On the other hand, AuNSPs-CA was taken up as a whole via an endosomal mechanism. These findings indicated that the surface ligands of AuNSPs could result in unique biological interactions (Diaz-Diestra et al., 2022).

The effects of carboxyl functionalised ultrasmall superparamagnetic iron oxide NPs (CU) or amine-functionalised ultrasmall superparamagnetic iron oxide NPs (AU) on the biological responses in human coronary endothelial cells were evaluated by Diaz et al. An increase in protein adsorption was observed for AU compared to that for CU after exposure to serum proteins, which indicated that a concentration-dependent decrease in cell viability and perinuclear accumulation occurred inside cytoplasmic vesicles after exposure to CU. Under non-cytotoxic conditions, the internalisation of CU was related to endothelial cell functional changes, as determined by a considerable decrease in the expression of endothelial-specific adhesion proteins and an increase in endothelial permeability. The researchers found that the surface chemistry of the ultrasmall superparamagnetic iron oxide NPs significantly affected protein adsorption and endothelial cell uptake, viability, and barrier function (Chamundeeswari et al., 2019).

Although carbon nanotubes have been used extensively in the field of medicine and biotechnology for several decades, they have several noticeable side effects. Luo et al. investigated the potential side effects of pristine multi-walled carbon nanotubes (P-MWNTs) and oxidised multi-walled carbon nanotubes (O-MWNTs) to assess specific macrophages *in vitro*. Their findings indicated that the effect of O-MWNTs on oxidative stress, cytotoxicity, and apoptosis of the unique macrophages was negligible, whereas, P-MWCNTs induced significant cytotoxicity, reduced cell viability, and promoted apoptosis (Luo et al., 2016).

## 5 Protein corona

The effects of the size, shape, chemical composition, or surface modification of NPs on their biological effects had been described and summarized previously (Table 1). However, another important effect of NPs could not be ignored. When NPs were exposed to the physiological environment as contrast agents or drug carriers (Kumar et al., 2018), their surfaces adsorbed various proteins, polysaccharides, and lipids. As proteins were the most abundant, the NP-biomolecular complex was collectively called protein corona (Li et al., 2022). Protein corona can change the characteristics and biological effects of NPs, such as transport mechanisms inside cells (Shaw et al., 2016). Therefore, further studies on protein corona *in vivo* might provide valuable information. The size, shape, and material composition of NPs might also affect the formation of protein corona (Wang et al., 2022).

**TABLE 1:** The size, shape, chemical composition, or surface modification of NPs on their biological effects.

Influence the results	The way it enters the cell	Oxidative stress	Mitochondrial function/Membrane potential	Membrane permeability	Organ accumulation
Influence factors					
Size	(Xia et al., 2019]	Zapor (2016)			
	Yang et al. (2022)	Li et al. (2018)			Xia et al. (2019)
	Zhang et al. (2021)	Li et al. (2022c)		Zapor (2016)	(Deng et al.. 2019)
	Sharma et al. (2022a)	Manke et al. (2013)	Zapor (2016)	Feng et al. (2022)	(Wang ct al., 2022a)
	Sharma et al. (2022b)	Sies and Jones (2020)			
	Zhao and Stenzel (2018)	Hanson and Batchelor (2022)			
Shape				Cho et al. (2010)	Ma et al. (2013)
	Joshi et al. (2022)			Soenen et al. (2010)	Luo et al. (2018)
	(Shao et A, 2017)	None	Nunes et al. (2022)	Spadea et al. (2022)	(Mutes et al., 2022)
	Champion and Mitragotri (2006)			Tree-Udom et al. (2015)	Talamini et al. (2017)
				Champion and Mitragotri (2006)	Dell'Aglio et al. (2023)
Chemical Composition or Surface Modification		Bai et al. (2020)			
	(Rai et al., 2020)	Luo et al. (2016)			
	Diaz-Diestra et al. (2022)	Manke et al. (2013)	(Alunad et al., 2017)	Chamundeeswari et al. (2019)	None
	Chamundeeswaii et al. (2019)	Ahmad et al. (2017)			
		Kongseng et al. (2016)			

Madathiparambil et al. synthesised two kinds of mesoporous SiO_2_ NPs with the same chemistry, porosity, and surface potential but different shapes (rod-shaped and spherical). The results showed that a significantly large amount of plasma and serum proteins was absorbed by the rod-shaped NPs than by the spherical NPs. The researchers speculated that the shape of the NPs played a key role in attracting specific proteins used by the immune system to recognise and clear foreign entities (Madathiparambil Visalakshan et al., 2020).

Zhang et al. synthesised three kinds of MWCNTs (original, MWCNTs-COOH, and MWCNTs-PEG) and investigated the effects of surface chemistry in the formation of a protein corona and the effect of the protein corona on cell uptake, cytotoxicity, and cell response. They found that the ability of the original MWCNTs to adsorb BSA and IgG was the strongest, whereas MWCNTs-PEG had the weakest ability to adsorb. Also, due to differences in the surface modification of chemical groups, the formation and composition of the protein corona were different. Proteins quickly coated the surface of the original MWCNT and MWCNT-COOH and made them more compact than MWCNT-PEG, and these compact forms were more effective in protecting cells from the MWCNT surface. Further investigation showed that hydrophobicity was the main reason for the increase in the cellular uptake of the original MWCNTs. However, the protein corona filled with dysoposnins was the major reason for the low uptake of MWCNT-COOH in RAW264.7 cells. The protein corona on MWCNT reduced the exposure of the surface to the medium and limited interactions with cells. As a result, fewer ROS or inflammatory cytokines were produced. Hence, protein binding played a key role in reducing toxicity (Zhang et al., 2019).

Some studies have also shown that the number and types of proteins adsorbed on the surface of NPs are closely related to the properties of NPs. Li et al. synthesised several NPs with similar shapes, sizes, and surface characteristics but different elasticity (45 kPa–760 MPa). After mixing these NPs with mouse plasma, they found that the composition of the protein corona formation changed non-monotonically with the increase in the elasticity of the NPs (Li et al., 2022). For example, apolipoprotein AI was more likely to form the corona protein over NPs of intermediate elasticity (75–700 kPa). Cheng et al. found that changing the components of lipid nanoparticles (LNPs) can make LNPs absorb specific proteins in the blood and target specific organs (Cheng et al., 2020).

**FIGURE 4:**
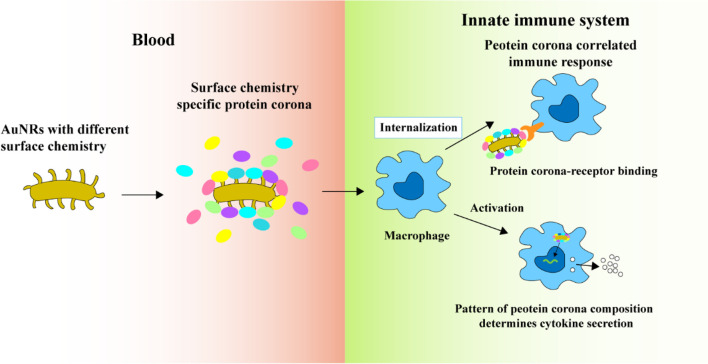
The protein coronas on AuNRs modified with various surface ligands of different chemical compositions and charges (Cai et al., 2020). Copyright 2020 American Chemical Society.

The research team undertook a study to ascertain the relationship between the ligand-protein corona and its biological effects, utilizing Pearson’s correlation coefficient analysis. They delved into the impact of the protein corona on how particles are internalised and the subsequent macrophage immune response. Their findings revealed that the surface chemistry of AuNRs played a role in determining the protein corona’s composition. Furthermore, the uptake of AuNRs by macrophages was predominantly steered by the interactions between the corona proteins and specific receptors present on the cell membrane, as shown in Figure 4. Notably, while surface charge did have some bearing on the protein corona, its influence was substantially lesser compared to that of surface chemistry (Cai et al., 2020).

**FIGURE 5:**
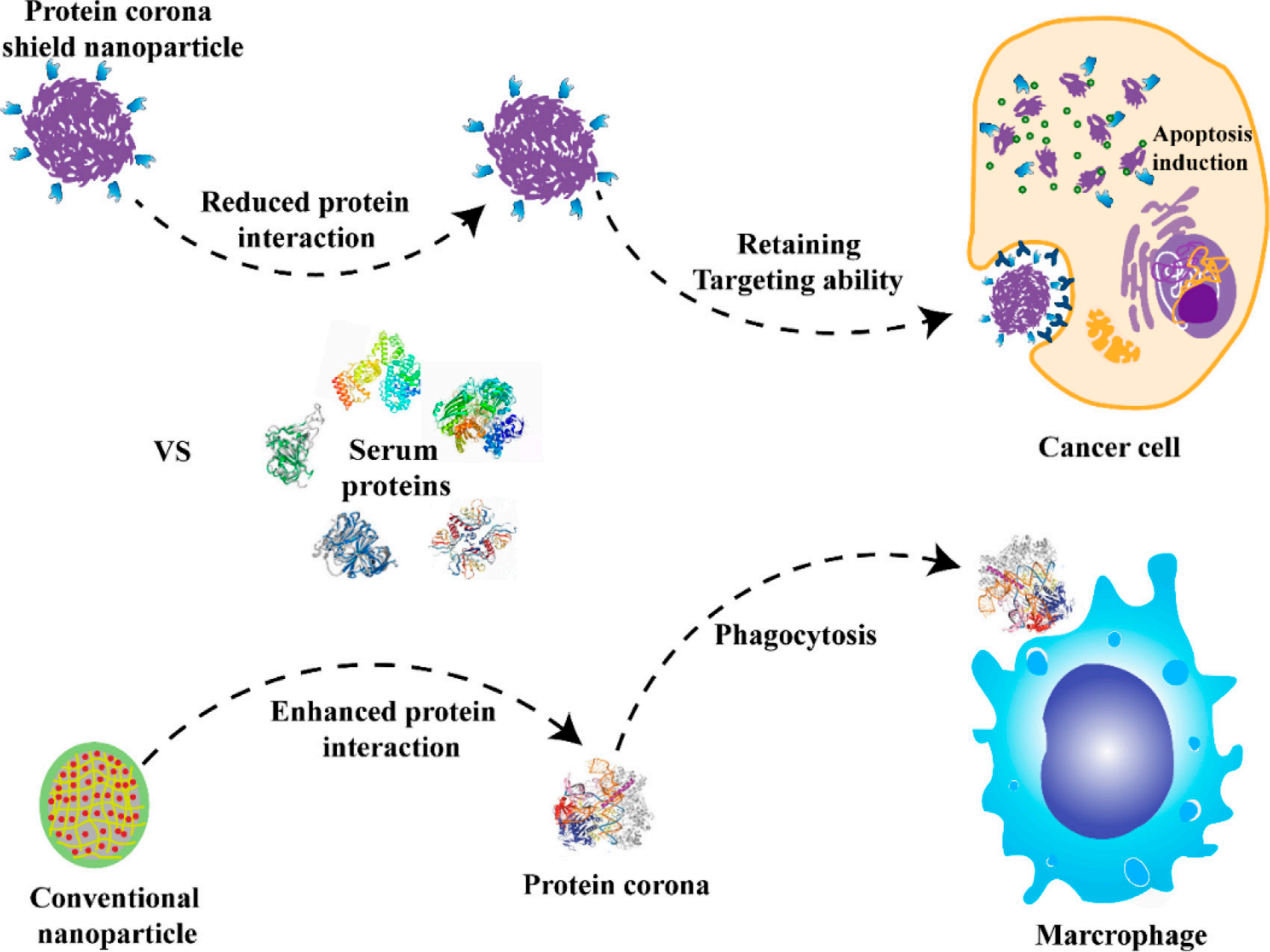
NPs with a preformed hard corona made of HER2 affibody, which helps maintain the targeting while reducing the PC formation and avoiding macrophage uptake compared with conventional NPs (Oh et al., 2018). Copyright 2020 Springer nature.

Salvati et al. found that the protein corona formed in the bronchoalveolar fluid increased the adsorption of macrophages to NPs by 2–5 times (Salvati et al., 2013). The formation of the protein corona promoted the absorption of NPs and led to the proinflammatory response of macrophages, and it also blocked the interaction between ligands and receptors (Mirshafiee et al., 2013), which in turn decreased the targeting efficiency (Zhao et al., 2021). To ensure the specific delivery of NPs within the bloodstream and to deter the undesirable effects of protein corona formation, the scientists introduced a novel targeting method. This method entailed attaching NPs to pre-coated supramolecular recombinant fusion proteins, as depicted in Figure 5. The sticky part of this fusion protein has a specific affinity for glutathione-S transferase, which subsequently connects to the HER2 receptor. By achieving thermodynamic stability in the intended orientation, the attached fusion protein acts as a protective corona. Such a corona can mitigate interactions with serum proteins, effectively thwarting the macrophage-driven removal of the NPs from the bloodstream. This strategic approach guarantees that the targeting remains precise both in laboratory settings and within living organisms (Oh et al., 2018). The NPs used in this research were shielded with a protein corona layer, limiting serum protein binding while preserving their inherent ability to target specific sites.

The formation of a protein corona can not only change the biological characteristics and targeting of NPs but also change the properties of proteins. Zhao et al. (2005) showed that human serum albumin (HSA) was the main binding protein in blood, and the AgNPs could adsorb HSA to form a stable protein corona on the surface of AgNPs. Using spectroscopic techniques, the researchers showed that the secondary structure and esterase-like activity of the protein were greatly impaired. Biomolecular and cell evaluation methods need to be combined to identify the harmful effects of the conformation of the protein corona by changing the protein which might provide insights into the interaction between NPs and biological interfaces, and thus, reduce the toxicity of the protein corona.

Fibrinogen is a key protein that participates in the protein corona composition of many types of NPs. Its conformational changes are crucial for activating the immune system. Electrostatic and π-π stacking interactions help in stabilizing the interaction between the fibrinogen and single-walled carbon nanotubes (SWCNTs). A study showed that the protein corona can reduce cytotoxicity (Martinez-Vargas et al., 2023) without affecting the biodegradation of SWCNTs in activated inflammatory cells. The findings laid the foundation for designing safer and less toxic NPs (Lu et al., 2018). Investigating the effect of NPs on protein conformation may not only help in effectively utilizing the properties of proteins to reduce the toxicity of NPs, but it may also reveal the mechanism of toxicity of NPs. Therefore, elucidating the mechanism of formation of a protein corona and the mechanism underlying the interaction between NPs and proteins *in vivo* is necessary.

**FIGURE 6:**
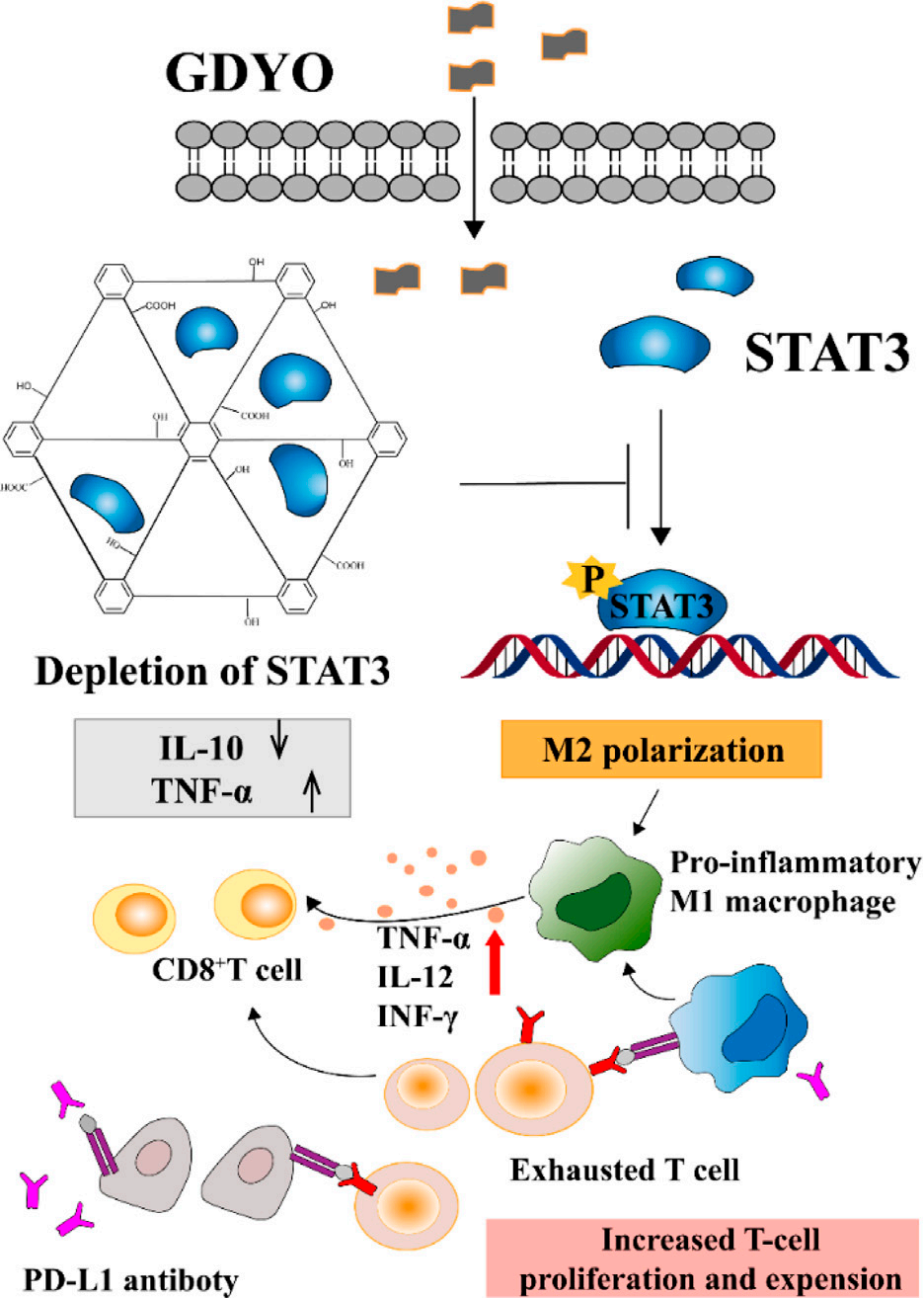
Proteins associated with the STAT pathway (Guo et al., 2021). Copyright 2020 American Chemical Society.

The manner in which various types of NPs influence macrophage polarization depends on distinct mechanisms. It's the structure and function of the protein corona formed within macrophages that steer the phenotype of the macrophage, as shown in Figure 6. In one particular study, it was observed that graphene oxide has the capability to restrain the immunosuppressive characteristics of tumor-associated macrophages. It achieves this by engaging with an intracellular protein, the signal transducer and activator of transcription 3 (STAT3). Notably, STAT3 is instrumental in tumor development and in directing the polarization of tumor-associated macrophages. The potent bond between graphene oxide and STAT3 hinders STAT3’s movement into the nucleus. This blockade fosters the emergence of a macrophage phenotype that is more inclined to inflammation. This research underscores the significance of tailoring NPs and fine-tuning the makeup of the protein corona since such modifications can profoundly impact the biological reactions triggered by NPs. It is crucial for researchers to factor in these insights during their experimental designs (Guo et al., 2021).

The above examples indicated that the formation of the protein corona also depends on the properties of NPs. It can also greatly promote the clinical application of NPs if its properties are utilised.

## 6 Summary and outlook

Although the field of biomedicine has progressed significantly, the application of NPs has several limitations, which hinder its further development. One reason is that the mechanism of interaction between NPs and biological systems is unclear. Some active mechanisms might produce biological toxicity, which researchers are investigating. The toxicity of NPs is closely related to their unique physical and chemical properties. However, some of these physical and chemical properties make NPs more applicable than traditional materials. Therefore, determining ways to elucidate the mechanism by which NPs impart toxic effects and balance the relationship between unique properties and toxicity is necessary. NPs can elicit an immune response after entering the body. Oxidative stress, inflammatory response, and influence of cell signalling pathways are the mechanisms through which NPs enter organisms and produce toxic effects. Finally, these factors can impair gene expression and promote cell death via autophagy. For tumour cells, hypertoxicity can cure cancer. However, ways to reduce side effects on normal cells need to be determined.

Due to the complexity of cells, organisms, and NPs, a simple overview cannot completely describe the biological mechanism of NPs, but it can provide new research directions. To increase the applicability of NPs in the field of biomedicine, we presented some suggestions.

(1) While selecting materials, new NPs with very low toxicity that can cause very little damage to biological cells need to be developed, to reduce the toxic effects of the material. Some NPs derived from organisms, such as exosomes, are widely used. However, limitations such as the low yield of exosomes and difficulty in their isolation and purification hinder their clinical applications. Researchers continue their search for new materials with low toxicity and ways to improve the existing ones.
(2) Generally, NPs with the most reasonable size and shape are designed and synthesised. Such NPs can not only pass through the biological barrier efficiently but can also be rapidly metabolised by the body. Thus, NPs can be used for diagnosis and treatment without the problems of having residues in the body.

Although NPs are widely used in various fields, scientists lack an understanding of the mechanism of action of NPs at the organ, tissue, cellular, and molecular levels (Li M. et al., 2022); however, information on these aspects might be the key to the clinical transformation of NPs. The safety and toxicity of NPs are issues of public interest. However, studies in the field of nanotechnology have focused on investigating the toxic dynamics of NPs (absorption, distribution, metabolism, and excretion), cellular uptake, and transport (Bondarenko et al., 2021). Biological mechanisms, including intracellular oxidative stress, inflammatory response, the disintegration of NPs, regulation of cell signalling pathways, and the formation of a protein corona (Yang et al., 2021), are important and might be the main topics of future research. Therefore, understanding the interaction between NPs and biological systems is necessary, and the reduction of the cytotoxicity of NPs is required for the advancement of nanotechnology. Through a comprehensive study of these basic elements, the mechanism by which NPs exhibit toxic effects might become clearer, which might lay the foundation for further application of NPs in clinical practice and benefit humans.
